# Raman Imaging and Fluorescence Lifetime Imaging Microscopy for Diagnosis of Cancer State and Metabolic Monitoring

**DOI:** 10.3390/cancers13225682

**Published:** 2021-11-13

**Authors:** Lucas Becker, Nicole Janssen, Shannon L. Layland, Thomas E. Mürdter, Anne T. Nies, Katja Schenke-Layland, Julia Marzi

**Affiliations:** 1 Department for Medical Technologies and Regenerative Medicine, Institute of Biomedical Engineering, University of Tübingen, 72076 Tübingen, Germany; lucas.becker@uni-tuebingen.de (L.B.); shannonlayland@yahoo.com (S.L.L.); katja.schenke-layland@uni-tuebingen.de (K.S.-L.); 2 Cluster of Excellence iFIT (EXC 2180) “Image-Guided and Functionally Instructed Tumor Therapies”, University of Tübingen, 72076 Tübingen, Germany; anne.nies@ikp-stuttgart.de; 3 Dr. Margarete Fischer-Bosch Institute of Clinical Pharmacology, University of Tübingen, 72076 Tübingen, Germany; nicole.janssen@ikp-stuttgart.de (N.J.); thomas.muerdter@ikp-stuttgart.de (T.E.M.); 4 NMI Natural and Medical Sciences Institute at the University of Tübingen, 72770 Reutlingen, Germany; 5 Cardiovascular Research Laboratories, Department of Medicine/Cardiology, David Geffen School of Medicine, UCLA, Los Angeles, CA 90073, USA

**Keywords:** Raman microspectroscopy, fluorescence lifetime imaging microscopy, tissue diagnostics, tumor metabolism, in situ imaging, 3D in vitro models

## Abstract

**Simple Summary:**

In spite of significant improvements in diagnosis and treatment options, cancer treatments still suffer from late detection and patient-specific multidrug resistance causing limited efficacy of systemic drug therapies. This review delineates the pioneering works on current advances in Raman spectroscopy and fluorescence lifetime imaging microscopy and their non-invasive application in in vitro and in vivo cancer identification, early-stage monitoring, drug screening, and metabolic analysis. In addition, the remaining challenges and limitations of the methods will be discussed and important aspects of their application in the clinic, enabling them to be a real asset to clinicians in the future and complementing current gold standard methods, will be explained.

**Abstract:**

Hurdles for effective tumor therapy are delayed detection and limited effectiveness of systemic drug therapies by patient-specific multidrug resistance. Non-invasive bioimaging tools such as fluorescence lifetime imaging microscopy (FLIM) and Raman-microspectroscopy have evolved over the last decade, providing the potential to be translated into clinics for early-stage disease detection, in vitro drug screening, and drug efficacy studies in personalized medicine. Accessing tissue- and cell-specific spectral signatures, Raman microspectroscopy has emerged as a diagnostic tool to identify precancerous lesions, cancer stages, or cell malignancy. In vivo Raman measurements have been enabled by recent technological advances in Raman endoscopy and signal-enhancing setups such as coherent anti-stokes Raman spectroscopy or surface-enhanced Raman spectroscopy. FLIM enables in situ investigations of metabolic processes such as glycolysis, oxidative stress, or mitochondrial activity by using the autofluorescence of co-enzymes NADH and FAD, which are associated with intrinsic proteins as a direct measure of tumor metabolism, cell death stages and drug efficacy. The combination of non-invasive and molecular-sensitive in situ techniques and advanced 3D tumor models such as patient-derived organoids or microtumors allows the recapitulation of tumor physiology and metabolism in vitro and facilitates the screening for patient-individualized drug treatment options.

## 1. Introduction

Cancer diagnosis and treatment remain one of the greatest challenges in medicine. The common theory regarding the genesis of cancer is that a primary tumor develops for a long time at the subclinical or microscopic level before spreading distantly and forming metastases [1]. To be clinically diagnosed, a tumor must reach a size of about 1 cm^3^ and contain about 10^9^ cells [2]. Consequently, there is a high probability that metastases have already spread at the time of diagnosis. Accordingly, there is an urgent need for new technologies that can detect the presence of tumor cells before the disease becomes clinically identifiable by the current diagnostic tools.

In recent years, the potential of optical and vibrational spectroscopic techniques for in situ and non-invasive cancer diagnosis has sparked a growing interest in clinical spectroscopy. Techniques such as Raman microspectroscopy and fluorescence-based methods, which are based on the study of the interaction between different types of radiation with matter and are able to evaluate and visualize molecular and biochemical tissue features, have significant applicability in early disease detection, intraoperative surgical guidance, and cancer therapy development [3]. Furthermore, such techniques offer the potential to acquire information about the molecular environment and the metabolism of cells. As a reprogrammed metabolism is considered a hallmark of cancer, this question of great interest can be addressed with optical techniques that can help to investigate and track tumor metabolism. In accordance with the “Warburg effect”, an aerobic glycolysis metabolism is a distinctive feature of cancer cells. Tumor cells require the consumption of surrounding nutrients to meet their energy and biomass needs [4,5]. The alteration of the metabolism is accompanied by a redistribution of nicotinamide adenine dinucleotide (NADH/NAD^+^) and flavin adenine dinucleotide (FADH_2_/FAD) ratios and the change from free to bound states [6,7]. Fluorescence lifetime imaging microscopy (FLIM) has proven to be an extremely sensitive method to detect such minute metabolic changes of these endogenous fluorophores and is therefore a powerful tool to assess the metabolic state of cancer cells. Compared to FLIM imaging, Raman microspectroscopy additionally provides label-free identification and localization of more than a defined number of fluorophores. Biomolecules such as proteins, lipids, or nucleic acids are Raman-active [8] and thus provide molecular fingerprints that are highly sensitive and can reflect a specific tissue state or cellular phenotype [9,10,11].

Given the increasing importance of Raman spectroscopy and FLIM, the purpose of this review is to explain the most promising modalities of these techniques for cancer imaging, metabolic monitoring, and diagnosis. In particular, we present the pioneering studies on Raman spectroscopy, coherent anti-Stokes Raman spectroscopy (CARS), surface-enhanced Raman spectroscopy (SERS), FLIM-FRET, and endoscopic devices for early-stage disease diagnosis, tissue border detection, drug monitoring, and the tracking of cancer metabolism.

## 2. Raman Microspectroscopy and Imaging

### 2.1. Physical Principle and Background

Molecules consist of two or more bonded atoms that are in perpetual electronic, vibrational, rotational, or translational motion. When laser light interacts with a molecule a variety of events can occur, including photon absorption, elastic or inelastic light scattering through the stimulation of vibrational modes within the sample, or the light may not interact and pass straight through the molecule [12]. Spectroscopy studies these interactions and depending on the applied wavelength, the molecule responds in different ways. Pure rotational transitions are induced at low energies in the microwave and far infrared region, whilst rotational–vibrational transitions appear in the infrared range [13]. Electronic transitions, on the other hand, appear in the visible and ultraviolet range of the electromagnetic spectrum and are accessed in UV/Vis spectroscopy [13]. While polar molecules can be accessed by emission or absorption spectroscopy, non-polar molecules are only accessible by the spectroscopy of scattered photons.

In the event of absorption, the energy of the incoming photon is equivalent to the energy gap between the ground state and the excited state of a molecule and leads therefore to the promotion to a higher energy state [14]. Elastic light scattering such as Rayleigh and Mie scattering, where light interacts with molecules such as dust or water particles in the air or lipid droplets, maintains the wavelength of the incident light [15,16]. In contrast, in the rare case of inelastic light scattering, energy is transferred from the incident light to the molecule (Stokes scattering) or from the molecule to the scattered light (anti-Stokes scattering), changing the energy of the emitted wavelength. These shifts in wavelengths correspond to characteristic shifts in the energies of the rotational and vibrational states and are thus characteristic material properties [12].

Those inelastic light scattering events are called the Raman effect and were first observed by Chandrasekhara Venkata Raman in 1923 [17]. Raman spectroscopy detects the effect of inelastic light scattering of a sample after interaction with a light source [18]. The shifts in wavelengths are given as wavenumbers, which are determined by the difference of the inverse incident and inverse scattered light. This difference is referred to as Raman shift and is only connected to the energetic properties of the molecular vibrations and independent of the applied laser wavelength. Raman spectra represent a molecular fingerprint, highly specific for individual molecules and sensitive to their surrounding and three-dimensional (3D) conformation [19,20]. In biological applications, important information about organic components such as DNA, lipids, or proteins can be detected from the spectral fingerprint region between 600 cm^−1^ and 1800 cm^−1^ [21].

### 2.2. Instrumentation

**Figure 1:**
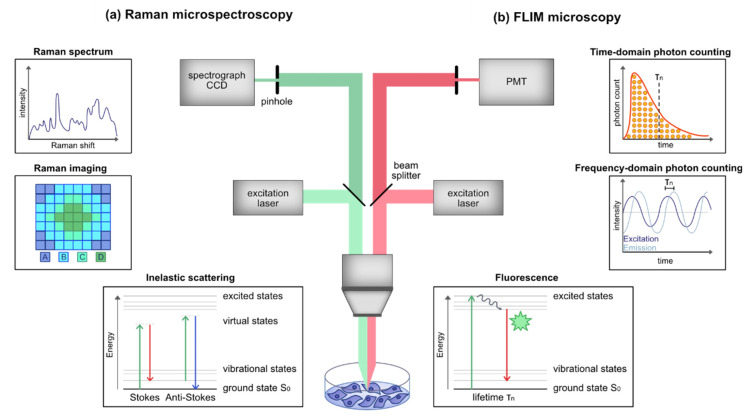
Schematic of Raman and FLIM instrumentation. The basic light pathway of both techniques is initiated at a laser source which is directed on the sample to induce photon–matter interactions. The emitted or scattered light is then directed through a beam splitter and a pinhole to the detector. (**a**) In Raman microspectroscopy, inelastic scattering results in energy transfer processes to higher (anti-Stokes) or lower (Stokes) energy levels than the excitation source. Raman spectroscopy requires a spectrograph e.g., with a charge-couple device (CCD) camera as a detector and Raman spectra are the typical readouts. By raster scanning, hyperspectral Raman images can be generated, where each pixel corresponds to one specific Raman spectrum. (**b**) Fluorescence lifetime decays are the targeted processes in fluorescence lifetime imaging (FLIM) microscopy. The average time that a fluorophore spends in the excited state can be detected by photomultiplier-tubes (PMT). The most common acquisition modes are frequency-domain or time-domain photon counting.

The common design of a Raman spectrometer comprises a laser source, a light pathway with filters and mirrors directing the laser to the sample and separating the inelastic scattering from other events such as elastically backscattered light from the laser, as well as a spectrograph with a detector (Figure 1a). Compared to basic Raman spectroscopy, Raman microspectroscopy includes an optical microscope setup to focus on or identify a certain region of interest within the sample via guidance by brightfield or fluorescence imaging. Single spectra acquisition or Raman imaging by scanning a defined region at a defined resolution can be performed with such setups and enable a high spatial resolution as well as the localization of certain components in heterogenous samples [22,23].

The basic instrumental setup in conventional Raman spectroscopy implies the generation and detection of spontaneous Raman scattering. Here, monochromatic continuous-wave laser sources are generally used. In contrary, Coherent-Anti-Stokes Raman spectroscopy (CARS) utilizes pulsed lasers that require exposure to higher peak powers for a proper signal-to-noise ratio [24]. The choice of the operating laser source and its wavelength depends on the scientific question that has to be addressed. In organic materials, shorter visible wavelengths and UV light cause strong photoluminescence and absorption, which may obscure the Raman signal or even damage the sample. Thus, a laser source within the visible or near infrared (NIR) wavelength range (500–1064 nm) is often more suitable for studying organic materials [12]. The intensity of the Raman signal is inversely correlated to the wavelength of the incident light. The Raman scattering at 532 nm, for example, is 4.7 times more efficient than at 785 nm [25]. Consequently, longer wavelengths of light require longer acquisition times to obtain good signal-to-noise ratios. In dynamic applications such as live drug monitoring, it is important to be able to perform fast measurements, otherwise the position of the analyte may change during a measurement. Therefore, the use of low wavelengths with shorter acquisition times is more suitable for live experiments than NIR. However, in fixed tissue samples or ex vivo body fluids, the usage of NIR lasers is an alternative and provides the advantage of deeper tissue penetration and less autofluorescence [26].

The spectrometer is a crucial component of any setup for performing Raman spectroscopy measurements. In a spectrometer, Raman scattered light is dispersed by a diffraction grating and directed to individual pixels of a detector so that each pixel reads out a small wavenumber range [27]. Depending on the targeted sample, the spectral resolution and the required spectral range need to be adjusted. If the sample investigation requires a high resolution of narrow spaced Raman peaks, a grating with a higher groove density is required to obtain a high spectral resolution. However, depending on the wavenumber range that should be covered, a high spectral resolution requires longer exposure times compared to less resolved Raman spectra [28]. Additional important parameters in resolution are the focal length of the spectrometer, the dispersion, and the size and quality of the used detector. The most common detectors are charge-coupled devices (CCDs). CCDs are silicon-based multi-channel array detectors suitable for UV, visible, and NIR light and consist of thousands or millions of individual detector elements (pixels). Being extremely sensitive to light, they are well suited for detecting the inherently weak Raman signal [29]. A more detailed explanation of all critical parameters and components in Raman spectroscopy instrumentation has recently been summarized by Ramya et al. [30].

### 2.3. Modifications

#### 2.3.1. CARS

CARS is a third-order nonlinear process which was first described by Maker and Terhune in 1965 and enables the weak spontaneous Raman signal to be enhanced [31]. In CARS setups, a pump laser with frequency ω_pump_ and a Stokes laser beam with frequency ω_Stokes_ are synchronized and illuminated on the sample to produce an anti-Stokes field signal Ω (1) corresponding to vibrational modes of the probed molecule [12,31].

Ω_anti-Stokes_ = 2 · ω_pump_ − ω_Stokes_ (1)

The signal of CARS is only detectable when the frequency difference between the pump and the Stokes beam matches the frequency of a Raman-active vibrational mode, otherwise there is no signal. In addition, the intrinsic coherent property enables the CARS signal to quadratically increase by the number of molecular vibrations in the focal volume [32]. In biological samples, CARS is very sensitive to lipid-rich structures when ω_pump_–ω_Stokes_ coincides with the symmetric CH_2_ stretching vibration at 2840 cm^−1^ and often enables faster data generation than in spontaneous Raman imaging [33,34]. An additional unique key advantage of the laser sources and setups applied in CARS is their ability to perform multimodal imaging. A typical CARS picosecond pulse excitation microscope is fully able to image CARS, sum frequency generation, and two-photon excitation fluorescence (TPEF) simultaneously [35,36].

#### 2.3.2. SERS

In the past decade, nanotechnological concepts have established themselves as a useful extension of conventional spectroscopy [37]. In surface enhanced Raman spectroscopy (SERS), the excitation of localized surface plasmon resonance at the surface of nanostructured metals such as gold or silver by light causes massively enhanced Raman signals from molecules in the immediate vicinity of the metallic substrate [38,39]. These metals can be designed in different shapes such as plates, rods, coatings on surfaces, or colloids [40,41]. Depending on the intended application, the SERS substrates can either be fixed to surfaces, liquids, or cells applied to them, or the nanoparticles can be dispersed in the sample. Readout modes range from the imaging and tracking of defined structures [42,43] to quantitative assays that yield comparable or superior sensitivity to conventional antigen-based assays [44,45,46]. However, the usage of such noble metallic nanostructures could lead to undesirable effects when used in cell experiments, since the plasmon excitation may interfere with catalytic reactions due to heat generation, providing energetic electrons and/or producing local string electromagnetic fields [47]. These constraints have been overcome successfully by coating the metallic nanoparticles with thin shells of chemically inert oxides such as SiO_2_ or Al_2_O_3_. This technique is then referred to as shell-isolated nanoparticle-enhanced Raman spectroscopy (SHINERS) [48].

Advantages of SERS techniques are their photostability, sensitivity and reduced tissue damage, since NIR lasers can be employed. Additionally, SERS can be used in combination with fluorescence spectroscopy. Here, SERS is favored due to its multiplexing capabilities by separating up to 10 different types of nanoparticles simultaneously using only one excitation wavelength [37,49].

## 3. Fluorescence Lifetime Imaging Microscopy

### 3.1. Physical Principle and Background

By the absorption of a photon, a molecule becomes electronically excited if the energy of the incident photon corresponds to the difference between two energetical states of the molecule, and can return to the ground state S_0_ via radiative (fluorescence or phosphorescence) or non-radiative processes (internal conversion, intersystem crossing, or vibrational relaxation) [14]. The fluorescence lifetime is defined as the average time that a molecule dwells in the excited state before returning to the ground state. Normally, the internal conversion is already completed before emission due to the typical fluorescence lifetime of about 10^−9^ to 10^−8^ s. For practical applications, the fluorescence lifetime is the time that elapses until the fluorescence intensity drops to the l/e of the value immediately after excitation [50]. The generation and detection of these fluorescence lifetime decays in biomolecules is the basic principle and readout of FLIM microscopy.

The advantage of FLIM compared to intensity-based fluorescence microscopy is the addition of the time component to fluorescence signals [51]. In conventional fluorescence microscopy, fluorophores can be distinguished from fluorophores with distinct spectral characteristics. On the basis of intensity, however, it is not possible to distinguish between fluorophores with similar spectra, nor to distinguish between individual molecular environments surrounding the same fluorophore [52]. FLIM is a powerful tool for the assessment of biophysical changes at molecular levels [53]. By measuring fluorescence lifetimes, it is possible to quantify chemical and physical changes to molecules including changes in temperature, viscosity, or pH [54,55,56,57].

### 3.2. Instrumentation

FLIM microscopy is usually performed in combination with a laser scanning or a wide field microscope setup. Conventional FLIM setups require a modulated or pulsed laser source for fluorescence excitation and imaging optics as well as sensitive optics capable to discriminate background photons, including tissue autofluorescence, from actual fluorescence lifetime signals (Figure 1b). In FLIM microscopy, two basic principles—time and frequency domain FLIM—can be distinguished. The most common setup for time domain FLIM is called Time-Correlated Single Photon Counting (TCSPC), which is utilized to determine the fluorescence lifetime of proteins and fluorophores. In TCSPC, the difference in time between the excitation of the sample by a pulsed laser and the arrival of the emitted photons at the photodetector is measured [58,59]. TCSPC requires a defined initial signal, which is provided by the electronics controlling the laser pulse, and a defined stop signal achieved by detection with single photon sensitive detectors (e.g., single photon avalanche diodes) [58]. Experimentally, the high-speed clock which measures the arrival time of emitted photons is implemented by an time-to-amplitude converter circuit converting the arrival time to an analog voltage which can be recorded [60,61]. Due to the statistical nature of time delays of fluorophore emission events, this measurement needs to be repeated many times resulting in time histograms [62]. For the generation of fluorescent lifetime images, the photons must be mapped to the distinct pixels accomplished by recording the absolute arrival time of the photons in addition to the relative arrival time with regard to the laser pulse [63].

The second approach to measure the fluorescence lifetime takes place in the frequency domain and is also known as the phase-modulation method [64,65]. In common practice, a sinusoidally modulated continuous wave laser is utilized for sample excitation. The fluorescence emitted by the sample is also sinusoidally modulated at the same modulation frequency as that of the excitation source and is analyzed in terms of its amplitude and phase shift. Compared to time domain FLIM, frequency domain FLIM allows for a faster imaging speed and does not require expensive pulsed lasers, but lacks performance at low photon counts [66,67]. A more detailed explanation of the instrumentation and description of further options for photon counting is out of the scope of this review and has been comprehensively described elsewhere [68].

### 3.3. FLIM Readouts

In biological samples, fluorescence lifetime decays can be obtained marker-independently or as probe-based FLIM readouts. Next to endogenously auto-fluorescent molecules, fluorescent dyes can be targeted and analyzed in regard to their fluorescence lifetime to gain insight into the metabolism or the composition of cells.

#### 3.3.1. Endogenous FLIM

In biomolecular research, FLIM is utilized to study the metabolic states of cells and tissue under several physiological conditions by exploiting the auto-fluorescent properties of the endogenous metabolic co-enzymes NAD(P)H and FAD [69,70].

Together, both co-enzymes are primarily involved in the mitochondrial tricarboxylic acid cycle and the electron transfer chain. Here, they are involved in the formation of ATP and reactive oxygen species (ROS) as parts of energy metabolism and the apoptosis pathway [71]. Additionally, NAD(P)H is also found in the cytosolic glycolysis pathway, which is important for high-proliferating cells such as cancer cells and is part of calcium homeostasis, gene expression, oxidative stress, and aging [72]. During oxidative phosphorylation, the produced NADH binds to its co-enzyme, leading to an increase in its fluorescence lifetime from less than 0.4 ns in the free NADH state to ~2 ns in its bound form [53]. Thus, according to the Warburg effect this leads to a substantial decrease in the fluorescence lifetime due to the shift in the metabolism to glycolysis, where less NADH and FADH_2_ are produced [5]. The ratios of free and bound co-enzymes (α1, α2) differ under different metabolic states [73] and therefore, both NAD(P)H and FAD have a great potential as biomarkers for their specific metabolic pathways [74].

#### 3.3.2. FLIM-FRET

Fluorescence resonance energy transfer (FRET) is an energy transfer phenomenon that has been used in many protein and nuclei analyses [75]. In FRET, a donor in the excited state can transfer some of its energy to an acceptor molecule [76]. To initiate these physical processes, several conditions must be met such as the distance between the donor and the acceptor of less than 10 nm with an overlapping spectrum, favorable dipole–dipole interactions, and a sufficient quantum yield [76]. If a donor molecule transfers its energy with increasing FRET efficiency, the fluorescence lifetimes of the donor decreases, which can be detected by FLIM [77]. The applications of FRET-FLIM mainly address at which location and within which timeframe protein–protein interactions occur in a cell [78].

#### 3.3.3. FLIM Probes

Similar to antibody-based fluorescence dyes, FLIM probes can be used to target specific molecules within cells or in their surroundings. FLIM probes can either be static [79], assuming their fluorescence lifetime does not change during experiments significantly, or become reactive [80]. While static FLIM probes are being utilized to track specific cell types or molecules [79,81], reactive FLIM probes respond to the cellular environment by changing their fluorescence lifetime. Reactive FLIM probes are designed to be sensitive to pH changes [82,83], the presence of electrolytes [84,85,86], oxygen [87,88], viscosity changes [89,90], and temperature changes [55,91] resulting in altered fluorescence lifetimes. Those responsive FLIM probes are also of particular interest in FLIM-FRET experiments. FLIM probes have the advantage of being less dependent on the fluorophore concentration and can thus minimize imaging artifacts and provide reproducible data over longer time periods. A more detailed overview of various FLIM probes can be found elsewhere [54,92].

## 4. Cellular and Tissue Diagnostics

Molecular-sensitive optical techniques such as FLIM and Raman microspectroscopy allow the simultaneous retrieval of information on the composition and the spatial distribution of a defined component in a sample. The marker-independent and non-destructive character of these techniques enable new readout approaches to improve cancer diagnosis.

### 4.1. Identification of Cancer Stages

In the progression of cancer, early-stage detection of the disease plays a vital role in increasing the recovery chances of patients. In the late stages of cancer and advanced metastasis there is huge lack of treatment options, and the survival rate decreases tremendously. The average 5-year survival rate of distant, late-stage breast cancer, where cancer has spread beyond the breast is at only 28% [93]. However, if diagnosed in the early stage, where the cancer is regional, the tumor can be removed surgically, and milder drug therapy is needed. The average 5-year survival rate for breast cancer diagnosed at an early-stage is 99% [93]. Therefore, the identification of cancer at the earliest stage is of great importance in cancer therapy. Up to date, only a limited amount of screening tests are available for a few cancer types. Those include, for instance, coloscopy, mammography, cervical cytology, or prostate specific antigen (PSA) testing, X-ray, histopathology magnetic resonance imaging or ultrasound [94,95,96,97]. Yet the effectiveness of some tests has been challenged; as the sensitivity and resolution are low, they remain potentially harmful or invasive and many patients do not adhere to medical guidelines for preventive screening [98].

Raman spectroscopy and FLIM have been utilized in the past decades in relation to several cancer types to explore the potential of these methods as minimally invasive screening tools for early-stage cancer detection. In the following paragraph, some of the most important cancer types are discussed by means of cancer stage detection. Particularly promising are the analyses of body fluids to identify biomarkers of the respective cancer stages as non-invasive preventive diagnostic approach and Raman histopathology, which requires no sample preparation and can be performed in vivo via fiber probe endoscopy or ex vivo during surgical intervention.

#### 4.1.1. Breast Cancer

To date, the diagnosis of breast cancer relies on histopathology, optical imaging (fluorescence or bioluminescence), magnetic resonance imaging, X-ray imaging or ultrasound. Histopathologically, early stage II breast cancer is difficult to diagnose unless a great number of biopsies are taken, and time-consuming histology protocols are necessary. Nargis et al. were able to differentiate between breast cancer stages II-IV by Raman spectroscopy of blood plasma samples [99]. With Raman spectroscopy and principal component analysis (PCA) it was shown that all clinically diagnosed breast cancer patients could be distinguished from healthy controls based on the spectral signatures of their blood samples. Blood samples from healthy volunteers exhibited higher spectral intensities at spectral bands such as 700 (amino acid methionine), 761 (amino acid tryptophan), and 1410 (COO_2_) cm^−1^ compared to blood samples from diagnosed patients. In contrast, the Raman spectral features at 1268 and 1285 (phospholipids), 1307 (CH_3_/CH_2_), and 1319 cm^−1^ (guanine (B,Z-marker) have higher intensities in diseased patient samples. Moreover, the progression of the disease could be tracked by elevated Raman signals at distinct wavenumbers in late stage IV, compared to previous cancer stages [99]. This approach of blood sample-based Raman spectroscopic analysis yields a more rapid analysis solution in the detection of early-stage breast cancer.

Recently, microcalcifications, considered as one of the first indicators of suspicious cancerous lesions were studied by Marro et al. [100]. Tissue sections of 26 patients with infiltrating ductal and lobular carcinomas with microcalcifications screened previously by mammography were subjected to Raman spectroscopy and multivariate curve resolution (MCR) combined with PCA analysis. They demonstrated the spatial distribution of DNA, lipids, proteins, cytochrome C and polysaccharides found in microcalcifications and showed that DNA is naturally encapsulated or adsorbed in calcifications present in invasive cancer tissues [100].

Abramczyk et al. employed Raman imaging to monitor changes in the redox state of mitochondrial cytochromes on breast and brain tissue, surgically resected specimens, and in vitro human brain cells of astrocytes [101]. They found that the vibrational mode of cytochrome C at 1584 cm^−1^ is the most prominent band and sensitive to changes in the redox state. In their experiments they demonstrated that Raman scattering is much weaker in the oxidized form of cytochrome C, concluding the peak at 1584 cm^−1^ originates from the reduced form of cytochrome C. In combination with the spectral band at 1634 cm^−1^, an important measure for the oxidized form of cytochrome C, they found parameters recapitulating the level of reduction and metabolic activity in cells. By comparing several grades of cancer progression (G1–G4), the intensity of the 1584 cm^−1^ peak increased with the aggressiveness of cancer up to G3, and then decreased for G4, leading to the conclusion that control mechanisms of the electron transport chain might be deregulated in cancers [101].

#### 4.1.2. Colorectal Cancer

Colorectal cancer (CRC) is the third most occurring cancer type in both women and men and the second cause of cancer mortality. An early prognosis in this type of cancer is crucial in the success of treatment of the patients, as the 5-year survival rate of patients in stage 1 and 2 is about 90%, but decreases to 11% at state 4 [102].

For diagnosis of tumor stages of CRC via Raman spectroscopy, various approaches targeting single cells, ex vivo tissue specimens or body fluids have been reported. In 2006, single living cells of the epithelium of CRC and control mucosa were observed by Raman spectroscopy [103]. PCA revealed a separation between both epithelial cells of mucosa and cancerous tissue according to spectral signals assigned to nuclei and proteins. With logistic regression classification, correct clustering was achieved at a sensitivity of 77.5% and a specificity of 81.3% [103]. In another study, classification of unstained paraffinized tissue sections of CRC yielded slightly higher classification results compared to living epithelial cells [104]. A diagnosis accuracy of 84.3% was detected using a partial least squares discriminant analysis. An advantage of this approach is that time-consuming cell culture protocols are not necessary. After deparaffinization, tissue slides can be directly placed under the microscope [104].

An alternative approach to detect CRC is the analysis of biofluids. The first experiments were conducted in 2011 by Li et al. who measured the serum samples of healthy donors and CRC patients [105]. Serum samples were placed in transparent tubes and subjected to Raman spectroscopy. By a principal component analysis and a linear discriminant analysis (PCA-LDA) classification model, they found a diagnosis accuracy of 88% with a sensitivity of 86% and a specificity of 97%. Advances in the analysis of biofluids were achieved with the development of new Raman spectroscopy-based techniques such as SERS. Lin et al. mixed blood serum from CRC patients with gold nanoparticles [106]. They placed the mixture in an aluminum chamber and excited it with a wavelength of 785 nm. SERS spectra yielded significantly higher spectral intensities compared to spectra without gold nanoparticles. While control blood samples exhibited higher intensities at 725 and 881 cm^−1^, cancerous blood showed higher signals at 494, 638, 823, and 1655 cm^−1^ [106]. With PCA-LDA, a classification sensitivity of 92.1% and specificity of 95.6% was obtained, which is higher than in the study by Li who used spontaneous Raman spectroscopy. Further improvements were pursued by the implementation of high throughput SERS platforms [107] and the development of a urine-based SERS test system for the identification of CRC at different progression stages [108]. Here, human urine samples of 63 diseased patients were analyzed before the patients received any chemotherapy. After the removal of cell debris, urine was mixed 1:1 with a gold nanoparticle solution and left to dry on an aluminum slide. By comparing spectral intensities, it was shown that healthy control urine samples exhibited statistically higher peak intensities at 725 and 1002 cm^−1^, while samples from cancer stage 1–4 showed higher intensities at 495, 640, 889, and 1358 cm^−1^. Stages 1 and 2 differed significantly from stages 3 and 4, enabling a distinction between early and late-stage CRC to be made [108]. A similar study was performed by Hong et al. using SERS on blood samples from CRC patients instead of urine [109]. Similarly, 1:1 dried mixtures of blood samples with gold nanoparticles were analyzed. Measurements at the edges of dried drops showed the best SERS signals due to the coffee ring effect enabling the identification of healthy and diseased states of CRC. Healthy donors showed higher signals of amino acids such as phenylalanine and tyrosine compared to CRC patients, whereas in diseased patients, lipid signals were statistically higher compared to controls at several spectral regions (1141, 1263 and 1484 cm^−1^). With machine learning tools such as PCA and support vector machines, the authors were able to classify both groups with accuracies of 90%, yielding a specificity of 100% [109].

**Figure 2:**
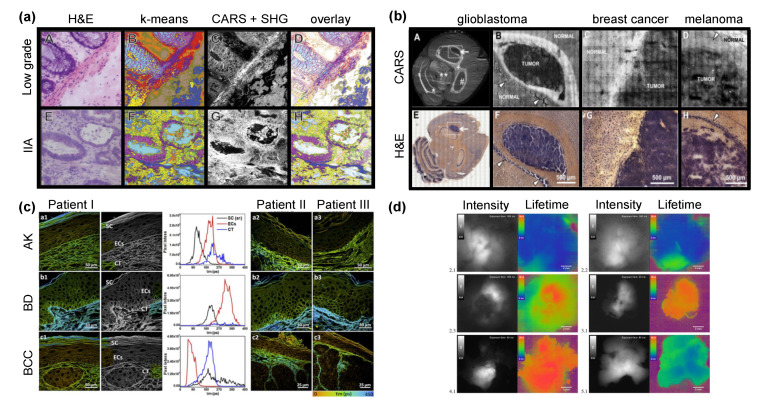
Application of Raman and FLIM imaging to discriminate tumor tissues. (**a**) Imaging of tissues from patients with low grade (A–D) and stage IIA (E–H) colorectal carcinoma. H&E staining (A, E), K-means clustering result of CARS spectra in the 2700–3000 cm^−1^ region (B, F), combined images of second harmonic generation (SHG) intensities at 408 nm and CARS signals at 2850 cm^−1^ (C, G) and overlay of k-means clustering with SHG and CARS (D, H) are shown. Reprinted with permission from [110]. (**b**) CARS (A–D) and H&E (E–H) imaging of mouse brain sections with an experimental human U87MG glioblastoma (A, B), breast cancer metastasis induced by MCF-7 cells (C) and melanoma induced by A375 cells (D). White matter of brain and normal tissue appears brighter in CARS images, while the tumor regions are darker. Reprinted with permission from [119]. (**c**) FLIM images of skin sections from actinic keratosis (AK), Bowen’s disease (BD), and basal cell carcinoma (BCC). The corresponding color-coding scheme ranges from red (0 ps) to blue (450 ps). For each patient and disease type, the mean fluorescence lifetimes (τm) of stratum corneum (SC, black line), epithelial cells (EC, red line), and dermal connective tissue (CT, blue line) were compared. Reprinted with permission from [124]. (**d**) 5-ALA fluorescence intensity (grey scale) and FLIM images (color) of brain tumor samples. Infiltrating tumor tissues characterized by increased lifetimes in green (2.1, 2.2), focal glial cell infiltration (2.3), strong 5-ALA positive fluorescence, according to increased lifetimes in red (3.1, 4.1) and glioblastoma with minimal infiltration of tumor cells are demonstrated (5.1). Areas in purple correspond to areas outside of the sample. Scale bars are equal to 2 mm. Reprinted with permissions from [125].

Furthermore, CARS imaging has been utilized to investigate CRC. In 2017, Petersen et al. used Raman spectroscopy and CARS as a method for virtual staining [110]. Hematoxylin & eosin (H&E) staining, the gold standard of medical diagnosis, was compared to label free CARS images of cryosections. An advantage of CARS in comparison to spontaneous Raman is the scanning speed which is close to video rates. Tissue slides were excited in the C-H region at 2850 cm^−1^ and subjected to k-means clustering methods after measurement to create pseudo color images. It was possible to identify the crypt, the connective tissue and the lamina muscularis by CARS imaging, showing similar structures as defined in H&E staining (Figure 2a). With their method, label free overview images of tissues were generated without the need for the histopathological staining of sections [110]. Apart from the acquisition of overview images, CARS can be applied to identify locations of lipid droplets at a higher spatial resolution. Recently, Guerenne-Del Ben et al. associated the increased expression level of Tropomyosin receptor kinase B (TrkB), a specific biomarker in the progression of CRC, with increased lipid droplet signals visible in CARS images [111]. They utilized three different CRC cell lines representing stages 1 to 3 of cancer progression and expressing different levels of TrkB. With CARS, cells were mapped at 2850 cm^−1^ and 2930 cm^−1^, representative for CH_2_ and CH_3_, respectively. Lipid droplets were visible as punctiform structures and could be associated with the cancer stage and progression of the cancer [111]. This result matched with studies by Geng et al. who showed significantly higher quantities of lipid droplets in high grade CRC and glioblastoma compared to low grade cancers and healthy normal tissue [112].

#### 4.1.3. Prostate Cancer

Screening for prostate cancer is routinely performed by the PSA test, which is the gold standard for a clinical readout. However, PSA screening has not been shown to decrease overall mortality and bears the potential risk of overdiagnosis or treatment complications [113,114]. To transfer Raman spectroscopic blood plasma analysis into the clinical routine, Medipally et al. built a rapid screening platform for liquid blood plasma samples [115]. A cover-glass test system was implemented to screen blood samples from prostate cancer patients and the spectral differences between diseased compared to healthy donors originated from lower beta carotene signals. Classification rates with a sensitivity of 96.5% when using exposure times of 40 s observed by PCA-LDA indicated the potential of the Raman spectroscopic test system to be used in prostate cancer diagnosis in the future [115]. Li et al. were also interested in prostate cancer as they utilized silver colloidal SERS nanoparticles mixed with blood serum of prostate cancer patients [116]. They found a diagnostic accuracy of 98.1% to differentiate between the control and diseased stages. To transfer SERS to the next step, Fu et al. developed a DNA-conjugated SERS nanotag to combine Raman spectroscopy with colorimetric lateral flow assays [117]. This nanotag binds to PCA3, a newly discovered biomarker for prostate cancer. With their technique they were able to detect PCA3 mimicking DNA concentrations in a range between 0.01 pM and 50,000 pM showing the sensitivity and potential of this method to be applied in early genetic disease recognition [117].

Similar to CRC assessment, prostate cancer biomarkers could not only be detected in blood or serum samples but in other body fluids such as the urine of prostate cancer patients. Urine samples were placed on a silicon waver with immobilized silver-nanoparticles. A multivariate analysis was employed leading to a 96.6% classification accuracy between recurrent and nonrecurrent patients after treatment [118].

#### 4.1.4. Brain Cancer

Spontaneous Raman-based probes suffer from low signal to noise ratios. To overcome this limitation, CARS was utilized by Uckermann et al. on cryosections of a murine brain (Figure 2b) [119]. Reduced CARS signals were found in tumor tissue sites compared to healthy white and gray matter. Even infiltration sites were clearly visible in the contrast of CARS images. In a later work, the same group utilized CARS on fresh human brain samples and in mice with brain tumors induced by human U87MG glioblastoma cells [120]. CARS images were generated at a wavenumber of 2850 cm^−1^ to access the lipid distribution in the tissues. In combination with TPEF and second harmonic generation imaging, false-color coded maps depicting the structure of the extracellular matrix, blood vessels and cell bodies were constructed. To identify tumorous lesions, 5-aminolaevulinic acid (5-ALA) was preoperatively administered. In their experiments, glioblastoma mice exhibited a reduced CARS signal in the tumor region compared to the surrounding tissue. Additionally, significantly increased nuclei diameters were observed in tumorous tissue. In comparison, normal cells in the gray matter of the brain showed less evident nuclei and lipid-rich cytoplasm [120]. The sensitivity of Raman spectroscopy compared to 5-ALA fluorescence-guided surgery in human glioblastoma was further investigated by Livermore et al., indicating the superior accuracy of the Raman-based approach [121]. Romeike et al. made an extensive study on 55 lesions of the central nervous system analyzing them with CARS and TPEF and found similar results as the Uckermann group [122]. With CARS and TPEF, information about the localization of tumors in brain tissue were obtained. Uckermann and Galli conducted a comprehensive study on 382 tumor patients and evaluated them with CARS [123]. Those promising results could be useful in clinical application, even though it is still necessary to miniaturize the CARS microscope for instance by development of miniaturized fiber lasers to be usable by surgeons.

### 4.2. Discrimination of Tumor Borders

Another important clinical need is the detection of cancer borders and cancer infiltrated zones during resection and surgeries. A fully resected tumor decreases the recurrence rate and increases the survival rate of the patient significantly [126].

In adrenocortical carcinoma for instance, one of the most aggressive solid tumors, characterized by a poor response to treatment and a high recurrence rate, the only possibility of long-term survival is enabled by complete tumor resection [127]. The same problem is described for breast cancer patients, where high rates of re-excision are needed, because a residual tumor is found in postoperative pathology analysis [128,129]. Frozen-section pathology is the gold standard utilized to assist in the resection of certain tumor types. However, for particular cancer tissues, such as breast tissue, frozen sections are challenging to accomplish as they contain high levels of fat and are prone to serious sampling errors, since only a small number of tissue sections can be rapidly mounted on microscope slides and examined by a microscope [130]. Here, Raman spectroscopy and FLIM offer useful complements in the guidance of tumor resection by overcoming the problem of sample preprocessing.

Multiplexed imaging is one approach used by scientists to recognize tumorous lesions on surfaces of fresh tissue. Wang et al. developed a rapid convection-enhanced topical staining device with SERS-coded nanoparticles for fresh breast tissue after a resection [131]. Five different SERS nanoparticles were applied topically on the surface of fresh tissues. They were designed to bind to EGFR, HER2, CD44, and CD24 and an untargeted control. With a spectral imaging probe, the fresh tissue was scanned. With this method, quantification of the expression of four biomarkers at the surface was achieved at a scanning speed of 5 cm^2^/min and a resolution of 500 µm. The whole process from staining until radiometric imaging takes only 5 min, making it a useful tool during resection to test if the whole cancerous lesions were removed [131].

The highest recurrence rate of all cancers due to inadequate resection occurs in bladder cancer [132,133]. Challenges in the resection of bladder cancer include invisible lesions during the operation. David et al. developed a SERS-based approach to visualize these invisible cancerous lesions [134]. Ex vivo human bladder tissue samples, cancerous and non-cancerous, were stained topically with SERS particles targeting CD47 and CA9. Passively targeted nanoparticles penetrated 5-fold deeper and bound to tumor tissue at 3.3-fold higher concentrations in cancer compared to normal bladder urothelium, suggesting the existence of an enhanced surface permeability and a retention effect in human bladder cancer [134]. In 2018, Bovenkamp et al. combined optical coherence tomography (OCT) with Raman spectroscopy for an improved staging and grading of bladder cancer. OCT was utilized as technique identifying the cancerous lesions. With guidance of OCT, Raman spectra of high- and low-grade bladder cancer were acquired at regions of interest and compared by PCA revealing a discrimination of cancer grade [135].

The potential of CARS being utilized in cancer discrimination has already been indicated in previous chapters. In recent research, CARS has been applied on brain tissue sections xenografted with glioblastoma cells and a living liver organoid model system to evaluate the potential of the technique even further. It was shown that CARS enables the discrimination between different cellular sub-populations based on its chemical content. This was correlated to the presence of sup-populations of organoids at different phases of the cell cycle, while in tissue sections to the tumor edge [136].

In addition to SERS and CARS, spontaneous Raman imaging is sensitive to identify cancerous tissues. Aubertin et al. classified fresh post-prostatectomy prostate cancer specimens with Raman spectroscopy. Leave-one-patient-out cross-validation showed a 90% classification accuracy between healthy and malignant areas of the resected tissue, indicating the potential of Raman spectroscopy to be utilized as a tool to confirm that all cancer margins have been removed completely [137].

FLIM microscopy has been successfully applied in the discrimination of tumor tissues, especially in the investigation of skin samples. In 2008, Galletly et al. employed FLIM to distinguish basal cell carcinoma from surrounding uninvolved skin in an ex vivo study [138]. They evaluated 24 unstained samples of Fitzpatrick skin types I and II with nodular, micronodular, superficial, and infiltrative skin cancer. Pseudo color FLIM images revealed that basal cell carcinoma (BCC) exhibited shorter fluorescence lifetimes compared to the surrounding healthy skin tissue [138]. Patalay et al. used two channel FLIM in 2011 to compare fluorescence intensities and lifetimes of benign naevi and malignant nodular BCCs [139]. Employing a 760 nm laser on fresh tissues, FLIM revealed statistically significant differences in both excitation channels <500 nm and >500 nm between naevi and nodular BCCs, representative of changes in NADH as well as melanin and flavin [139]. Morphological aspects of the cells were acquired by FLIM in 2012 by Seidenari et al. [140]. FLIM was combined with multiphoton laser tomography to assess the morphological hallmarks of BCC and healthy skin. They found an enlargement of the intercellular space and irregular contours in the BCC samples but not in healthy skin. Additionally, cells exhibiting longer fluorescence decay times were found in each BCC sample, while in healthy skin samples blue shifts were only visible in one-third of the samples [140]. In an in vivo mouse study, Miller et al. analyzed squamous cell carcinoma (SCC) by multimodal fluorescence imaging methods [141]. The endogenous fluorophores lipofuscin and flavin demonstrated shorter fluorescence lifetime decay curves in SCC compared to normal skin. The potential of screening for several diseases at once was explored by Luo et al. who employed FLIM as tool for identification of actinic keratosis, Bowen’s disease, and BCC (Figure 2c). FLIM images were compared with H&E and fluorescence stains and showed higher contrasts, underlining the ability of FLIM to be used as a complement to the gold standard procedure [124].

In 2010, wide-field FLIM was used by McGinty et al. to study ex vivo gastrointestinal tissues [142]. FLIM images clearly revealed differentiation between colonic adenocarcinoma and normal tissue which was compared to H&E staining. Edge detection of malignant lesions was possible by the increased fluorescence lifetime compared to non-cancerous areas. Similar observations were found in malignant oral mucosa cell lines as well as in cervical tissue when compared to non-malignant cells [143,144]. However, Skala et al. reported in an in vivo study that the fluorescence lifetime of normal oral epithelial cells is longer compared to precancerous cells [145]. The same results were found by Pastore et al. in melanomas of skin compared to healthy tissue [146]. Erkkilä et al., developed an imaging system combining a dual tap complementary metal-oxide semiconductor with wide-field FLIM allowing high speed imaging in the analysis of high- and low-grade gliomas [125]. They achieved scanning of a field of view of 11 × 11 mm at a 12 Hz imaging rate. Discrimination between cancerous lesions and healthy tissue was possible on differences in the fluorescence lifetime of protoporphyrin IX (PpxIX) induced by 5-ALA (Figure 2d). The same group, focused their work further on PpIX induced accumulations in glioblastomas indicating the potential of the method being utilized in clinical procedures [147].

Recent advances in Raman spectroscopy and FLIM have pointed out the benefits of these techniques to be implemented as an addition to gold standard methods for early-stage cancer detection as well as in the discrimination of cancer borders in tissue. However, in order to be applicable in daily medicine and to allow robust and comparable diagnostic results, standardized protocols are needed, especially for FLIM imaging which is highly sensitive to the physiological environment of the cells.

### 4.3. Endoscopy

Nowadays, early-stage cancer detection and edge detection of cancerous lesions is still challenging. Whether a suspicious change—for example a lump in the breast—contains cancer cells can only be determined with certainty on the basis of tissue or cell specimens. These are obtained by means of a biopsy or puncture and then examined microscopically by a pathologist in a histological examination. Depending on the organ and the location of the suspicious tissue area, different biopsy methods can be used: punching out a cylinder of tissue (punch biopsy), aspiration of tissue or cells (fine-needle biopsy, fine-needle puncture), or sampling of tissue with a scalpel (excisional biopsy) or endoscopically with tiny forceps [148,149,150,151]. It is also possible to obtain smear biopsies or body fluids and further examine cells, for instance on the cervix (Papanicolaou examination) [152]. However, these procedures are time-consuming and expensive.

As explained in the previous chapters, non-destructive optical analysis methods have great potential in the detection of cancer in ex vivo samples. Therefore, scientists have developed fiber probe-based Raman- and FLIM endoscopes that can be inserted into the instrument channel of medical endoscopes and placed in gentle contact with the tissue enabling in vivo tissue classification. This methodology offers the great advantage of in situ diagnosis and could potentially replace the resection of patient tissue for diagnostic purposes only.

#### 4.3.1. FLIM Endoscopy

In 2018, Jo et al. developed a multispectral time-domain FLIM handheld endoscopic device designed to capture autofluorescent emissions from collagen, NADH, and FAD [153]. To achieve this, collected fluorescence emission was divided into three emission bands at 390 ± 20 nm, 452 ± 22.5 nm, and >500 nm, respectively. In their pilot study, 73 patients with oral lesions such as cancer or dysplasia were involved as well as healthy donors and patients with benign inflammatory lesions. Prior to resection and histopathological evaluation, oral lesions and visually inconspicuous areas were scanned with the FLIM endoscopic device. Increased metabolism, representable by NADH/FAD ratios, was demonstrated to be a specific biomarker of dysplasia or cancer; however, the autofluorescence signal of collagen was not. With a developed machine-learning algorithm based on the FLIM images, the automated detection of early-stage oral cancer and mild dysplasia was achieved with 95% sensitivity and 86% specificity. Additionally, a separation to benign inflammatory conditions could be visualized by FLIM, demonstrating the potential for an in vivo diagnosis of oral cavity cancer, dysplasia, as well as benign inflammatory lesions which are usually difficult to distinguish visually from malignant lesions [153]. A similar study was conducted in 2020 by Duran-Sierra et al. who performed metabolic imaging of precancerous and cancerous oral lesions with a wide-field FLIM endoscopic system [154] and further advances in instrumentation and implementation of FLIM probes for endoscopy and intraoperative assessment were recently reported [155,156,157].

#### 4.3.2. Raman Endoscopy

Beginning in 2000, Shim and coworkers demonstrated an in vivo Raman endoscope system usable for colorectal cancer detection [158]. The central delivery fiber of their probe had a core diameter of 400 µm and was surrounded by seven collection fibers with a diameter of 300 µm. The smooth tip had a diameter of 2 mm and fitted in standard gastrointestinal endoscopes. The proposed sampling depth of their probe was around 500 µm, depending on the contact angle. First measurements with their probe proved the insensitivity to blood background interference and no detectable heat generation [158]. The same group intensified their study in 2002 to assess the diagnostic potential of their tool by measuring colon polyps [159]. Polyps were identified ex vivo and in vivo at an accuracy of 93% and 95%, respectively, using PCA-LDA.

In the following years, many groups started to work on endoscopic Raman devices. In 2011, Bergholt et al. managed to decrease the integration time to 0.5 s by utilizing 32 collection fibers [160]. In a follow up study, they measured Raman spectra at several colon sites such as transverse colon, descending colon, sigmoid and rectum of 50 patients [161]. By performing partial least square discriminant analysis (PLS-DA), they managed to separate cancerous tissue from normal tissue with an accuracy of 88%. Further developments have aimed to reduce the size of the collection fiber even more. In 2017, Petersen et al. manufactured a probe system with an excitation fiber at a width of 105 µm and collection fibers of 200 µm size [162].

In neurosurgery and brain cancer, the application of fiber probe-based Raman spectroscopy and CARS has been extensively investigated. In 2015, Desroches et al. built a Raman spectroscopy probe system usable for intraoperative brain tissue classification [163]. Classification of healthy and tumor-invaded brain tissues was performed with a boosted tree algorithm leading to a classification accuracy of 92%. Further brain studies were conducted by the same group and the experimental setup and readout were further evaluated and optimized. During open cranium surgery, a handheld detection system combining Raman spectroscopy, intrinsic fluorescence spectroscopy (IFS) and diffuse reflectance spectroscopy (DRS) was tested on patients with metastatic cancers and grade 2–4 gliomas. The combination of Raman spectroscopy with IFS showed an increase in classification accuracy, sensitivity and specificity up to 97%, 100%, and 93%, respectively [164]. Compared to the experiments with the combined system of Raman spectroscopy, IFS and DRS, core needle biopsy resulted in a classification accuracy of only 90% [165].

Successful fiber-optic Raman endoscopy was also conducted on several other tissue sites and organs including esophagus [166], bladder [167], oral cavity [168], breast [169], larynx/nasopharynx [170,171], lung [172], or cervix [173].

Other prototypes of Raman catheters for intravascular Raman spectroscopy were realized in addition to the developments of fiber-optic Raman probes for endoscopically accessible organs. Buschman et al. investigated the molecular composition of an artery wall utilizing a 2.5 mm thick Raman catheter capable of measuring forward and sideward [174]. In further developments, the size of such Raman catheters were further decreased to up to 600 µm and were used to analyze coronary atherosclerosis in vitro in a rabbit model [175] and in vivo in living rat’s esophagus and stomach [176]. Recent technical development includes the equipment of the endoscopic sensor part by a SERS substrate with 30 nm sized gold nanoparticles [177]. This device was tested on malignant oral squamous cell carcinoma, verrucous carcinoma, premalignant leukoplakia, and disease-free conditions and were classified with an accuracy of 97% [177]. Furthermore, a prototype for angle-resolved Raman spectroscopy was developed, incorporating an electromagnetic NdFeB micro rotary actuator to a Raman probe that enabled 360° measurements of peripheral lung bronchi in bronchoscopy [178].

Raman endoscopy has been proven to be a sensitive tool for monitoring diseased lesions in vivo; however, it does not offer the benefits of wide-field imaging visualizing suspicious areas guiding surgical procedures. Therefore, scientists have aimed to combine Raman spectroscopy with other imaging techniques such as white-light reflectance, narrow-band Imaging [179] or even with other spectroscopic methods such as DRS [180] or FLIM [181]. After the identification of a region of interest by a wide-field technique, the extent of the diseased lesions or early malignant transformations could then be addressed by the Raman spectroscopy module providing molecular characterization. With multimodal FLIM/Raman imaging rapid in vivo characterization of a rat brain was provided [181].

## 5. Monitoring of Metabolic Processes and Pharmacokinetics

In addition to early stage cancer diagnosis and the lack of reliable tools ensuring complete tumor resection, questions of tumor metabolism and personalized medicine are the focus of clinical oncology. As described previously, abnormal metabolism plays a crucial role in cancerous lesions and is of great interest for understanding tumorigenesis. Furthermore, personalized medicine is becoming increasingly important in modern medicine. Patient-specific failure of pharmacological therapies, e.g., due to multidrug resistance, encouraged numerous studies to test patients’ response to drug treatment in advance. In the following section, the advances of FLIM and Raman spectroscopy on those topics are summarized and evaluated, with a special focus on studies performed on in vitro tumor models.

### 5.1. In Vitro Tumor Models

**Figure 3:**
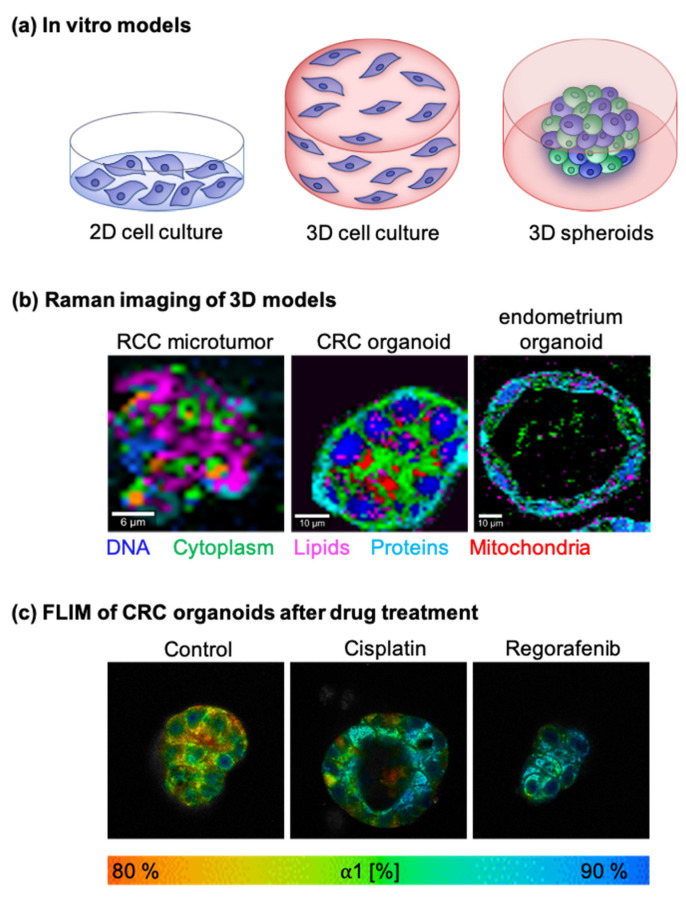
In vitro cancer models and Raman/FLIM imaging of 3D patient-derived tissue models. (**a**) Schematic illustration of 2D and different 3D in vitro tumor models. (**b**) Raman images of renal cell carcinoma (RCC) microtumors, colorectal cancer (CRC) organoids [218] and endometrium organoids. False color-coded intensity distribution heatmaps indicate DNA (blue), cytoplasm (green), lipids (pink), proteins (light blue) and mitochondria (orange/red). (**c**) Fluorescence lifetime imaging microscopy of CRC organoids. Displayed are the ratios of unbound to bound NADH (α1 [%]) of control organoids and organoids 24 h after cisplatin and regorafenib treatment. Patient-derived microtumor and organoid samples were provided by C. Schmees and A. Koch and retrieved in accordance with the Declaration of Helsinki; the protocol was approved by the Ethics Committee of the Medical Faculty at the University of Tübingen (150/2018BO2 and 379/2010BO2).

In vivo and ex vivo analyses of tissues or body fluids are highly relevant for the improvement of diagnostic approaches and the identification of novel cancer biomarkers, enabling the detection of malignant lesions at an early stage. Furthermore, a better understanding of cancer metabolism, the tumor microenvironment, and the tumor–immune interaction is in high demand in order to accelerate the efficacy of cancer therapy, especially on a patient-individualized aspect. In addition to in vivo models, comprehensive in vitro models can offer mechanistic insights into tumors as they are less expensive, easily accessible by molecular imaging techniques, and are constantly evolving in relation to the effective recapitulation of human physiology [182,183,184,185]. Consequently, this is where non-invasive techniques allowing real-time monitoring of cellular processes, tumor metabolism or drug kinetics, can be applied as in situ readouts for in vitro models. The selection of a suitable cell source and culture format is essential for the in vitro test system and approaches range from cell line-based two-dimensional (2D) systems to complex organoid-based microfluidic systems (Figure 3).

#### 5.1.1. Two-Dimensional Cell Cultures

Two-dimensional cell cultures have been the standard method to culture cells since the early 1900s and are a well-established strategy to perform in vitro cell-based studies. The first researcher to culture cells in 2D was Harrison in 1907 with cells originating from nerve fibers [186]. Since then, culturing methods have been improved in order to maintain the growth of primary cells in vitro [187,188], allowing the isolation and analysis of primary cell lines obtained from living organisms that closely mimic the genetic features of the corresponding tissue. However, this isolation is difficult, and the life span is often short in the 2D cell culture setting. As an alternative, bioresource centers, such as the ATCC (American Type Culture Collection), generated established cell lines and offer characterized models of various types of cancer cell lines that are routinely used in research [189,190]. Both primary and established cell lines have been used extensively in cell biology research and have been essential for the discovery and development of new drugs. Screening programs in well-characterized cell line panels such as the NCI-60 or the GDSC1000 cell line panel including thousands of compounds and more than 1000 human tumor cells have led to significant advances in cancer chemotherapy [191,192].

However, monolayer-cultured cells grown on a flat plastic surface gradually lose the tumor specific heterogeneity and do not adequately represent cells in vivo. The organizations of cells and the extracellular matrix have 3D arrangements in their natural state, which means that monolayer cell cultures do not accurately mimic in vivo cellular environments, including cell-cell and cell-matrix communications, nutrient status, and physiological or biochemical properties [193,194,195]. Due to many disadvantages of conventional 2D cultures, there is a need to develop more robust in vitro models capable of simulating in vivo solid tumors that may improve the efficacy of anti-cancer drug discovery and biological studies.

#### 5.1.2. Three-Dimensional Cell Cultures

In this context, 3D in vitro models are gaining increasing interest in the field of drug screening and cancer research, due to their improved ability to recapitulate the complexity of the tumor microenvironment (TME) including the tumors’ cellular heterogeneity and cell-cell interactions [196,197]. In fact, the reproduction of such characteristics in 3D promotes the formation of nutrient, oxygen, and signaling factor gradients, as well as the establishment of unique gene expression patterns similar to those observed in vivo in solid tumors [198]. Various 3D models have been developed, including tissue explants, spheroids and the recently expanding field of organoids, although the terms have been used interchangeably in the literature [199].

Spheroids were first introduced in the early 1970s by Sutherland and colleagues [200]. They can be formed by spontaneous aggregation of cells in the presence or absence of an extracellular matrix (ECM). Although spheroids are well-suited models to investigate intra-tumoral gradients of oxygen, nutrients, or drugs, they are arguably of low complexity in mirroring tumor organization [201,202]. In contrast, organoids can be cultured from embryonic stem cells, induced pluripotent stem cells (PSC) and adult stem cells (ASC) and do resemble the corresponding organ in terms of architecture, genetics and function, while heavily being reliant on the presence of an ECM [203,204]. Spheroids and organoid culture models have distinct and overlapping purposes and they differ in terms of tumor cell sources, protocol for culture and the time required for establishment. Detailed description of the differences and applications have been reviewed extensively elsewhere [205,206]. Although both spheroids and organoids promise greater representation of the primary tissue compared to cell lines, they both lack the original tumor architecture as well as the tumor specific tumor microenvironment. This limitation can partly be solved by co-culturing the primary 3D cell culture with a variety of cell types, including patient-derived immune cells or cancer-associated fibroblasts but still representing a simplified model of the situation in vivo [207,208,209,210,211].

#### 5.1.3. Other 3D Models

An ideal model should reflect the complex microenvironment and contain all cell types within the same 3D architecture, distribution, and ratio as they were present in a patient’s tumor. Tissue explants represent a cell culture method, whereby fresh, surgically resected tissue can be used immediately for drug studies without the deconstruction of the tumor. The techniques of tissue cultivation have been improved continuously since the use of tumor tissue explants to demonstrate glycolysis and formation of lactic acid published by Warburg in 1923 [212]. In the 1950s, different matrices (e.g., sponge) have been introduced to support the tissue explants [213]. To overcome the variability due to different dimensions and accelerate the preparation, Krumdieck et al. developed an instrument to prepare round tissue slices of a given dimension (e.g., 6 mm diameter and 300 µm thickness) still used by different investigators [214]. Although these models based on primary tissue have been shown to replicate characteristics and chemosensitivity profiles of in vivo patient tumors [215], they have not been widely accepted in the drug development pipeline to date. The reasons for this are not entirely clear but may be related to the burgeoning use of cell line, organoid and patient-derived xenograft model systems, all of which can be propagated for longer periods of time. However, the growing evidence of the importance of the tumor microenvironment and tumor heterogeneity, the development of patient-derived microtumors [216,217] and their integration in microfluidic devices as well as improvements in marker-independent imaging techniques such a Raman spectroscopy and FLIM might revive the explant approach.

### 5.2. Tumor Metabolism

A first study on the cellular metabolism by assessing fluorescence lifetimes of intrinsic NADPH and flavins was conducted in 1992 by Schneckenburger et al. on a yeast model [219]. Since then, numerous other studies have followed, especially in the field of cancer metabolism. Cong et al. used FLIM to compare the behavior of the breast cancer cell line 4T1 in an in vivo-like 3D model to 2D cell culture [220]. The authors examined the response to two novel drug candidates that target metabolism by inhibiting monocarboxylate transporters or target mitochondria and disrupt the oxidative phosphorylation. Their studies revealed that cell culture conditions show significant differences in the response to the compounds. Three-dimensional cells in a collagenous matrix formed anastomosing multicellular networks and spherical acini, while in the 2D cell culture epithelial plaques were formed in reaction to the compounds. Additionally, cellular NAD(P)H showed longer fluorescence lifetimes in the 3D matrix due to the increased population of enzyme-bound NAD(P)H [220].

FLIM probes such as tetramethylrhodamine methyl ester (TMRM) or fluorescent dyes are predicated to study the mitochondrial membrane potential, which has been shown to be hyperpolarized in cancer cells compared to normal cells [221,222]. Application on human colon cancer HCT116 cells and Lgr5-GFP mouse intestinal organoids allowed visualization of the heterogeneity of mitochondrial polarization during the cell cycle progression [223]. Translation of TMRM-FLIM allowed the metabolically active cells in the stem cell niche to be revealed and revealed the changes in membrane potential among Lgr5-positive and other proliferating and differentiated cell types [223].

With FLIM-FRET, Martin-Villar et al. studied the interactions between Podoplanin-eGFP and CD44-mRFP in SCC [224]. Expression of Podoplanin in tumor cells was linked to increased cell migration. FRET interaction with CD44-mRFP showed that Podoplanin is associated with the plasma membrane of migratory phenotypes [224]. Another FLIM-FRET approach is to track the up-regulation of RhoA activity levels, which are related to numerous cellular activities and are drivers of cell motility in cancer [225]. Pajic et al. applied an eGFP/mRFP Raichu-RhoA biosensor [226] and Nobis et al. developed an in vivo RhoA-FRET mouse expressing a RhoA-FRET biosensor from the Hprt locus [227]. They showed that RhoA activity can be co-opted in invasive metastatic mammary and pancreatic carcinomas. Additionally, they were able to directly visualize the inhibition of RhoA using small molecule inhibitors [227]. Metabolic changes in high- and low HER2-expressing SCC cell lines were assessed by Miller et al. in 2015 by dual channel FLIM [228]. Metabolic changes were chemically induced and detectable by changes in fluorescence lifetime distribution and intensities.

Another promising target for FLIM-FRET measurements is caspase-3 which is a crucial component in the apoptotic pathway [229]. Activated by death-inducing signals, capsase-3 is cleaving a number of important cellular proteins. In gene expression studies, caspase-3 transcript and caspase-3 protein expression were found to be absent in 75% of cancerous tissues, indicating the importance of this type of protease in cancer development [230]. In 2015, Xiao et al. utilized genetically-encoded caspase-3 FRET reporters to study drug induced apoptosis in breast cancer cells [231]. Keese et al. encoded the caspase-3 FRET sensor in a mouse model [232]. They engineered murine colorectal cancer cells that express caspase-3 activity FRET sensors which were then injected into mice. As a result, peritoneal and liver metastasis was induced in mice. Their model allowed in vitro, post mortem, in vivo and ex vivo analyses of chemotherapy-induced apoptosis by monitoring the caspase-3 activity in the cancerous lesions [232].

Coban et al. used FLIM-FRET technology to monitor the metabolic effects of drugs on live HCC1954 epithelial breast cancer cells [233]. Cells were transfected with plasmids containing GFP-tagged DEP-1 phosphatase. Tyrosine kinase inhibitory effects of gefitinib and phosphatase-based manipulation of EGFR phosphorylation were analyzed 24 h after transfection. They discovered a drug-induced increase in the stability of EGFR homodimers, which could have a biological impact on the proliferation potential of cancer cells [233]. EGFR/HER2 dimerization after drug treatment was studied by Waterhouse et al. in paraffin embedded cancer cells revealing the potential to access any receptor dimerization in tissues [234].

Tumor metabolism can not only be accessed in the tumor cells themselves, but also the tumor microenvironment plays an essential role in metabolism and responsiveness to drug treatment [235,236]. As most cancer cells produce ATP by glycolysis, cancer microenvironments are shifted to lower pH values, reforming the extracellular matrix for cancer metastasis [237]. FLIM was utilized by O’Donnell et al. in 2018 to monitor the cancer milieu by quantitative measurement of pH in engineered tissues [238]. They fused a cellulose-binding domain with a pH-sensitive enhanced cyan fluorescent protein. HCT116 human colon carcinoma cells were incorporated in the scaffold and subset to the protonophore FCCP. Their FRET pair was sensitive to increases in extracellular acidification by a decline of 0.2–0.4 pH units.

### 5.3. Drug Monitoring

Drug metabolism and uptake, dose-dependent efficiency and developed drug resistances are important measures in the treatment of cancer patients. In the last decade, it has been recognized that these parameters are highly variable among patients which requires personalized treatment options [239,240,241,242]. Screening of dose-dependent efficacy of anti-cancer drugs prior to treatment increases the success rate and prevents drug overload and can thus avoid additional suffering of the patients. In addition, therapies could be made more cost-effective through such patient-specific information.

Spectroscopic- and fluorescence-based approaches are of great interest in clinical oncology, as they have the potential to trace the uptake and metabolism of anti-cancer drugs in cells. In the following paragraph the current advances are briefly summarized.

#### 5.3.1. Raman Spectroscopy-Based Drug Monitoring

Defined chemical substances such as drugs exhibit highly specific Raman spectra which enable infiltration tracking into cancer cells by Raman spectroscopy.

5-Fluorouracil (5FU) is an anticancer drug commonly used in systemic chemotherapy but is also used to treat non-melanoma skin cancer topically [243]. Alkylcarbonyl prodrugs of 5FU have been synthesized that penetrate deeper into tissue compared to 5FU [244]. Zhang et al. were able to track the penetration depth of 5FU with help of Raman spectroscopy as they topically applied 36 mM of 1-ethyloxycarboyl-5FU and 22 mM of 5FU to skin tissue from white, hairless Yucatan pigs for 20 h [245]. Spectral images were acquired at 5 µm increments. Factor analysis in the range between 800 and 1015 cm^−1^ revealed that the tracking of both pro-5FU and 5FU is possible based on higher signal intensities in the defined spectral range compared to untreated skins. Additionally, a factor analysis was able to differentiate between both drugs according to their different spectral features [245].

In 2009, Harada et al. studied the uptake and conversion of CPT-11 (irinotecan) to SN38, both topoisomerase I inhibitors in HeLa human cervical cancer cells, MCF-7 human breast cancer cells and mitoxantrone-resistant MCF-7/MX cells [246]. Cells were treated with 100 µM CPT-11 for 120 min prior to measurements. It was shown that in MCF-7 cells incubated with CPT-11, a distinct signal appeared at 1561 cm^−1^, correlating to the drug signal. In mitoxantrone-resistant MCF-7/MX cells, however, this signal was not detected, indicating poor uptake or fast metabolization [246]. Erlotinib, a drug targeting the tyrosine kinase binding domain of the EGFR receptor, was studied by El-Mashtoly et al. in 2014 [247]. Raman spectroscopy was applied to detect the spatial distribution of erlotinib in human colorectal adenocarcinoma SW480 cells. After 12 h of incubation with 100 µM erlotinib, cells were fixed with 4% paraformaldehyde and subjected to label free Raman spectroscopy. The drug was tracked inside the cells based on its specific spectral signature within the range between 2085 and 2140 cm^−1^, assigned to its C≡C triple bond. A comparison of the raw erlotinib spectrum to the spectrum of erlotinib tracked inside cells demonstrated spectral alterations in the region between 1170 and 1595 cm^−1^ indicating metabolic processing.

Another tyrosine kinase inhibitor, neratinib, was studied in 2018 by Aljakouch et al. [248]. HER2-positive breast cancer cells (SK-BR-3) and NSCLC cells were utilized and subjected to 5 µM neratinib for 8 h. They were able to monitor neratinib according to its C≡N stretching appearing at 2208 cm^−1^. Fluorescence images revealed that neratinib and its metabolites accumulated in lysosomes of cells.

#### 5.3.2. SERS-Based Drug Monitoring

As dissolved drugs in therapeutic concentrations yield only low spectral intensities, the group of Quiao developed a smart nanoparticle-based system increasing the sensitivity of drug monitoring experiments [249]. They manufactured a gold-silica nanocarrier incorporating doxorubicin, a model anti-cancer drug. As a test platform, HeLa and HEK293 cells were used which were incubated for 4 h with silica-gold nanoparticles. SERS images recorded at the intensity of 1078 cm^−1^ revealed the distribution of the drug nanocarrier system in a significantly higher sensitivity compared to fluorescent staining of the same cells [249]. In 2013, Huang et al. incorporated doxorubicin and silver nanoparticles in a graphene oxide-based nanoplatform [250]. They employed graphite flakes which were oxidized according to Hummer’s method into graphene oxide. SERS experiments were performed 2 and 6 h after incubation of the graphene oxide based nanoplatform on human cervical carcinoma cell lines. In cells, doxorubicin was dissolved from the complex by π–π interaction between the drug and graphene oxide due to the acidic environment. Graphene oxide could be identified by SERS imaging at the 1326 cm^−1^ band. According to the recorded images graphene oxide was located in the cytoplasm of the cells but not in the nucleus. To track the release of doxorubicin, spectral maps were created at 460 cm^−1^ and compared to 1595 cm^−1^, characteristic for graphene oxide. After 2 h doxorubicin was still on the graphene oxide surface, determined by the overlapping SERS maps. Time series revealed decreasing mean SERS signals after 24 h, revealing a decreasing amount of doxorubicin at the graphene oxide surface. Fluorescence staining revealed that the release of doxorubicin takes place in the lysosomes of cells [250].

#### 5.3.3. FLIM-Based Drug Monitoring

The potential of FLIM being used to monitor drugs in cells has also been investigated. Dai and coworkers encapsulated doxorubicin with a grafted pseudopeptide consisting of poly(L-lysine adipamide) with poly(ethylene glycol) side chains (PLyAd) [251]. They monitored the uptake of the micellar-encapsulated doxorubicin over 72 h via endocytosis into small vesicles in the cytoplasm with negligible nuclear accumulation. Romero et al. encapsulated doxorubicin with Poly(lactide-co-glycolide) (PLGA) nanoparticles and monitored the uptake in HepG2 cells [252]. Fluorescence lifetimes of doxorubicin in cells were demonstrated to be shorter (1.61 ns) than inside the PLGA nanoparticles. The presence of only one lifetime led to the conclusion that doxorubicin was completely released inside the cells [252]. In 2018, Saari et al. utilized Orgeon green labeled anti-cancer drug paclitaxel, which was loaded onto exosomes and microvesicles [253]. FLIM microscopy showed that exosomes deliver drugs by endocytosis, while microvesicles enter cells either by endocytosis or fusion with cell membranes.

## 6. Challenges and Limitations

This review has given an overview of the current advances of Raman techniques and FLIM-based analyses to improve early stage and in situ cancer diagnosis and to extend the mechanistic understanding of the tumor metabolism. Both Raman spectroscopy and FLIM are very sensitive tools that can monitor the microenvironments and metabolic states of biological specimens. In particular, recent advances in SERS experiments combined with body fluids represent easy-to-perform, non-invasive methods to detect early cancer progression. Since cancer prognosis is highly dependent on complete tumor resection, improving rapid and sensitive detection is of great importance. Here, Raman spectroscopy and FLIM offer great potential, especially with the development of endoscopic devices, offering non-destructive in vivo molecular analyses. Besides direct clinical application, Raman spectroscopy, FLIM, and their modifications offer significant value on understanding cancer progression, as they allow for the in situ screening of metabolic changes in cells in real-time. In addition, the prospective application in the field of personalized medicine should be emphasized. Combined with rapidly evolving physiologically relevant 3D in vitro tumor models such as organoids, microtumors, and their integration on microfluidic platforms, these imaging modalities would allow for personalized high throughput testing on patient tissues or cells to support treatment decisions. The readouts and scenarios that could be modeled on these platforms would not only be limited to access drug response or tumor metabolism but also further treatment options such as efficacy of radiotherapy can be evaluated and predicted via Raman spectroscopy of individual patient tissues [254]. Consequently, these setups can provide a tremendous improvement in the patient’s quality of life and increase treatment efficacy.

Despite all the benefits, challenges remain in relation to both techniques that must be tackled in the future, especially regarding their translation into clinics.

In FLIM microscopy, standardized protocols are in high demand, since FLIM is influenced by and sensitive to many factors such as the confluence of cells, pH, temperature and the inter-/intra-cellular heterogeneity, which is already challenging in in vitro setups but even more relevant in vivo [255]. The non-specific binding of FLIM probes, which can influence the result, is still problematic [256]. Molecular probes are also subject to high background signals in vivo due to autofluorescence [257]. Designing FLIM probes with longer lifetimes may prevent these effects [258]. Here, the usage of quantum dots with long fluorescence lifetimes may be an alternative [259]. Another challenge in using FLIM-FRET probes arises in experiments where the probes are overexpressed by cells. Here, there is a risk that disturbances of protein–protein interactions may occur, as the natural state of the cells may be disturbed [260].

For the broad clinical implementation of FLIM, application in large samples is particularly necessary. Here, wide-field FLIM and recent advances in establishing macroscopic FLIM imaging have been achieved, allowing the scanning of whole tissue samples [142,261].

Most of the drawbacks of Raman spectroscopy refer to the weakness of the Raman effect, which leads to prolonged measurement times. In this respect, the potential damage to the tissue by the laser must be considered and balanced in correlation to spatial and spectral resolution, image size, and acquisition time [262]. Laser damage due to heat generation can be avoided, for example, by hydrating the sample and using water immersion objectives [263]. Another limiting factor regarding acquisition speed is the selection of the laser source. In biological experiments, especially in in vivo studies, the use of light sources in the NIR wavelength range (e.g., 785 nm) is better suited than shorter wavelengths (e.g., 532 nm), as this avoids problems of tissue autofluorescence and allows for deeper tissue penetration depths [264,265]. However, excitation in the NIR requires longer acquisition times compared to visible light. Here, signal enhancing techniques such as CARS imaging or stimulated Raman scattering allow for video-rate imaging of predefined spectral bands and represent alternatives to spontaneous Raman imaging [266,267]. For all methods, however, overcoming the effects of ambient light is still challenging, as measurements would need to be performed in a dark room environment or light has to be blocked from entering the specimen [268,269].

The final goal of most cancer-related research questions is their translation into clinics. First of all, safety aspects have to be considered. The possibility of sterilization and the reusability is of great importance for endoscopic systems. Here, the material of the instruments must be designed in a way to withdraw corrosive effects of sterilization media. The design and manufacturing of disposable fiber-optic Raman probes for endoscope systems is still technically challenging [270]. Moreover, laser-based imaging instruments must be safe to use for both, patients, and clinicians. Depending on the laser class, specific personal protection equipment, a controlled work environment and training, as well as defined architectural conditions of the individual facility are required according to the Code of Federal Regulations (21 CFR 1040.10) [271]. In particular, usage of lasers on patient tissue is regulated by the ANSI guidelines for eye/skin exposure [272]. Additionally, the instruments should not disrupt or interfere with the current gold standard treatment protocols. Especially, in fluorescence-based clinical setups the influence of room light and other illumination sources need to be considered. Finally, one of the main key points include a rapid data visualization allowing clinicians to make good decisions in real-time. In both, FLIM and Raman imaging instrument handling is more intuitive and easier to learn than a thorough understanding and interpretation of the resulting data. Therefore, the instruments need to perform quick data assessments and analysis. Here, first approaches report on the implementation of automated and machine-learning based classification and readout options [273,274,275], which will be an inevitable task in the future, especially when multimodal imaging approaches and conventional imaging tools will be combined in the surgery room.

## 7. Conclusions

In this review, recent achievements, and future challenges of non-invasive biophotonic techniques were summarized. FLIM microscopy and Raman spectroscopy show very promising results in obtaining a deeper understanding of cancer metabolism, drug efficiency and uptake as they allow for time-resolved monitoring. Moreover, clinical cancer diagnosis could highly benefit from in situ histopathology and the identification of novel cancer biomarkers in body fluids. Results of classification and accuracy rates achieved by Raman spectroscopy and FLIM are highly encouraging to allow for the further evolution and implementation of these tools for application in routine clinical setups.
